# Sex Differences in Dopamine Receptor Signaling in *Fmr1* Knockout Mice: A Pilot Study

**DOI:** 10.3390/brainsci11111398

**Published:** 2021-10-24

**Authors:** Anlong Jiang, Le Wang, Justin Y. D. Lu, Amy Freeman, Charlie Campbell, Ping Su, Albert H. C. Wong, Fang Liu

**Affiliations:** 1 Campbell Family Mental Health Research Institute, Centre for Addiction and Mental Health, Toronto, ON M5T 1R8, Canada; anlong.jiang@camh.ca (A.J.); le.wang@camh.ca (L.W.); jy.lu@mail.utoronto.ca (J.Y.D.L.); amy.freeman@medportal.ca (A.F.); charlie.campbell@mail.utoronto.ca (C.C.); ping.su@camh.ca (P.S.); albert.wong@utoronto.ca (A.H.C.W.); 2 Department of Pharmacology, University of Toronto, Toronto, ON M5S 1A8, Canada; 3 Institute of Medical Science, University of Toronto, Toronto, ON M5S 1A8, Canada; 4 Department of Psychiatry, University of Toronto, Toronto, ON M5T 1R8, Canada; 5 Department of Physiology, University of Toronto, Toronto, ON M5S 1A8, Canada

**Keywords:** Fragile X syndrome, sex difference, L-stepholidine, dopamine signaling, *Fmr1* knockout, D1 receptor, D2 receptor

## Abstract

Fragile X syndrome (FXS) is an X-chromosome-linked dominant genetic disorder that causes a variable degree of cognitive dysfunction and developmental disability. Current treatment is symptomatic and no existing medications target the specific cause of FXS. As with other X-linked disorders, FXS manifests differently in males and females, including abnormalities in the dopamine system that are also seen in *Fmr1*-knockout (KO) mice. We investigated sex differences in dopamine signaling in *Fmr1*-KO mice in response to L-stepholidine, a dopamine D1 receptor agonist and D2 receptor antagonist. We found significant sex differences in basal levels of phosphorylated protein kinase A (p-PKA) and glycogen synthase kinase (GSK)-3β in wild type mice that were absent in *Fmr1*-KO mice. In wild-type mice, L-stepholidine increased p-PKA in males but not female mice, decreased p-GSK-3 in female mice and increased p-GSK-3 in male mice. Conversely, in *Fmr1*-KO mice, L-stepholidine increased p-PKA and p-GSK-3β in females, and decreased p-PKA and p-GSK-3β in males.

## 1. Introduction

Fragile X syndrome (FXS) is the most common inherited cause of intellectual disability and autism, caused by mutation of the fragile X mental retardation (*FMR1*) gene that leads to insufficiency of the fragile X mental retardation protein (FMRP) [1,2]. FXS includes a spectrum of clinical manifestations, ranging from learning disabilities, attention deficits and hyperactivity to severe intellectual disability with autistic symptoms [3,4,5]. Current treatment for FXS consists of therapy for speech, physical, or behavioral problems, [6,7] and medications for FXS-associated seizures, mood dysregulation, hyperactivity, and attention deficits [8,9]. Better and more specific biological treatments targeting FXS disease mechanisms are needed [10,11].

FXS results from mutations in the fragile X mental retardation 1 (*Fmr1*) gene located at chromosome Xq27.3 that encodes FMRP. The most common *Fmr1* mutation leading to FMRP deficiency is a trinucleotide repeat expansion, consisting of a CGG in the 5′-untranslated region (5′-UTR). Normally there are 6–54 repeats [12] and >200 repeats is considered a mutation. The trinucleotide expansion triggers the methylation of CGG sequences and the *FMR1* promoter along with deacetylation of associated histones and chromatin condensation [13,14,15,16] ultimately resulting in epigenetic transcriptional silencing and decreased FMRP. Low levels of FMRP are also associated with various other mental health diseases including schizophrenia, bipolar disorder and major depressive disorder [17].

The prevalence of FXS has been estimated as 1.4 per 10,000 males and 0.9 per 10,000 females [18,19], and this significant sex difference is consistent with an X-chromosome linked disorder [20]. FXS-associated attention deficits, hyperactivity, anxiety, and autism are less severe in females, [21] who consequently have better overall outcomes and quality of life [22]. Sex-specific behavioural abnormalities are also observed in *Fmr1*-KO mice, in motor coordination, social interaction, learning, memory, and anxiety-like and repetitive behaviours [23]. Male *Fmr1*-KO mice are hyperactive compared with females, consistent with the hyperactivity and attentional deficits seen in boys with FXS [24,25]. Male *Fmr1*-KO mice have more rearing behaviour and less ultrasonic vocalizations compared to females [26] while female *Fmr1*-KO mice have more repetitive behaviours, impaired response inhibition and better motor coordination.

One functional pathway that could explain the attention deficits and hyperactivity in FXS is the dopamine system. FMRP regulates how dopamine modulates AMPA glutamate receptor subtype 1 (GluR1) surface expression through the D1 receptor [27]. In *Fmr1*-KO mice, there are fewer D1 receptors [28] that are hyperphosphorylated and hyperactivity is rescued by D1 receptor agonists. Thus, we hypothesized that other aspects of dopamine signaling related to the dopamine system could be abnormal in the *Fmr1*-KO mouse. In addition to classical dopamine receptor signaling through G-proteins, the dopamine D2-like receptors also regulate protein kinase B (Akt) through beta-arrestin 2 and glycogen synthase kinase 3β (GSK-3β) [29,30], which we examined in this paper.

We chose to modulate dopamine receptors with L-stepholidine, a natural compound derived from *Stephania intermedia* [31] that is both a D1 receptor agonist and D2 receptor inhibitor [32]. *Stephania intermedia* is a plant used in traditional Chinese medicine, and L-stepholidine has been studied as a potential antipsychotic agent. L-stepholidine can attenuate morphine-induced conditioned place preference [33] and has neuroprotective effects against memory deficits caused by chronic methamphetamine exposure [34]. In addition, L-stepholidine can improve memory and synaptic plasticity in Alzheimer’s disease models through D1-mediated PKA signaling [35].

## 2. Materials and Methods

### 2.1. Animals

All procedures were approved by the local Animal Care Committee at the Centre for Addiction and Mental Health (CAMH), Toronto, Canada, following guidelines by the Canadian Committee for Animal Care. C57Bl/6 wild-type mice were purchased from Charles River Laboratories (Wilmington, MA, USA), and breeding pairs of *Fmr1*-KO mice (with C57Bl/6 background) were purchased from the Jackson Laboratory (B6.129P2-*Fmr1*^tm1Cgr^/J, Stock No: 003025), and bred at the CAMH animal facility. Animals were acclimated to our facility for one week prior to the start of experiments.

Animals were housed at 20–23 °C with a 12-h day-night cycle (7 AM-7 PM). All animals were fed by standard Laboratory Rodent Diet 5001 (LabDiet, St. Louis, MO, USA), and the feed was available in the feeder above the cage on a free choice basis.

### 2.2. Drug Treatment

L-stepholidine was dissolved in sterile dimethyl sulfoxide (DMSO, Sigma-Aldrich, Burlington, MA, USA) at 1 mg/mL and stored at −20 °C. Prior to injection, stock solutions were diluted in filtered phosphate-buffered saline (PBS). Ten-week old *Fmr1*-KO and wild-type mice were injected intraperitoneally (*i.p.*) with L-stepholidine at a dosage of 10 mg/kg for seven consecutive days; control animals received PBS only. On the day following the last injection, mice were sacrificed by cervical dislocation, and the whole brain was removed for further analysis.

### 2.3. Total Protein Isolation and Sample Preparation

Brain tissue was homogenized on ice and total protein was extracted in a buffer containing 50 mM Tris-Cl, 150 mM NaCl, 2 mM EDTA, 1% NP-40, 0.5% sodium dodecyl sulfate 0.5% sodium deoxycholate, 1% Triton X-100, protease inhibitor cocktail (1:100; Sigma-Aldrich, Burlington, MA, USA) and phosphatase inhibitor cocktail (1:100; ThermoFisher Scientific, MA, USA), at pH 7.4. Samples were shaken at 4 °C for one hour followed by centrifugation at 10,000× *g* for 10 min. Protein concentration was quantified by bicinchoninic acid (BCA) assay. After equalization of protein concentrations, samples were denatured at 90–100 °C for 10 min in Laemmli buffer (Bio-Rad) supplemented with 5% β-mercaptoethanol (Sigma) and then subjected to Western blot analysis.

### 2.4. Western Blot

Equal amounts of protein were loaded onto gels subjected to SDS-PAGE (sodium dodecyl sulfate-poly-acrylamide gel electrophoresis), followed by transfer to a nitrocellulose membrane. The membrane was blocked with 5% non-fat milk for one hour at room temperature and then incubated with the primary antibody: anti-PKA (1:2000, rabbit, Cell Signaling Technology, Danvers, MA, USA), anti-phosphorylated PKA (Thr-197) (1:2000, rabbit, Abcam), anti-GSK-3α (1:1000, rabbit, Cell Signaling Technology), anti-GSK-3β (1:1000, rabbit, Cell Signaling Technology), or anti-phosphorylated GSK-3α/β (Ser-21/Ser-9) (1:1000, rabbit, Cell Signaling Technology), overnight at 4 °C. The membrane was then incubated with the appropriate horseradish peroxidase conjugated secondary antibody diluted in 1% bovine serum albumin in Tris-Buffered Saline (TBS) supplemented with Tween-20 for two hours at room temperature. The proteins were visualized by ECL clarity reagents (Bio-Rad) or enhanced chemiluminescence reagents (Amersham Biosciences, Piscataway, NJ, USA). The blot images were collected using the Bio-Rad ChemiDoc MP imaging system (Bio-Rad), and the intensity of the bands quantified by Image Lab software (Bio-Rad).

### 2.5. Statistical Analysis

Results are presented as the mean ± standard error of the mean (SEM). The statistical tests were performed using GraphPad Prism 9 (La Jolla, CA, USA). Before conducting statistical analyses, the normality of data was confirmed using the Shapiro-Wilk test. The student’s *t*-test (unpaired, two-sided) was used to compare two groups and one-way ANOVA followed by Tukey’s multiple comparisons test was used to analyze multiple groups.

## 3. Results

### 3.1. Fmr1-KO Animals Have Reduced Dopamine D1 Receptor-Mediated Signaling

**Figure 1:**
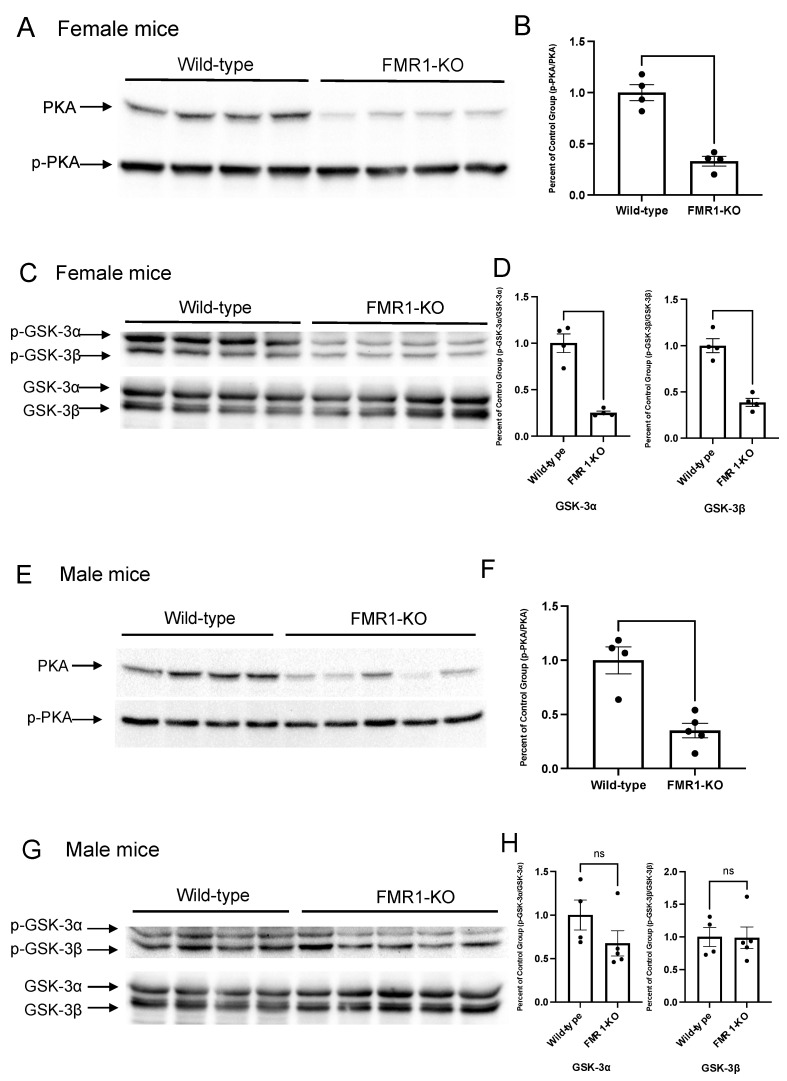
*Fmr*1-KO altered the dopaminergic signaling pathway. Ten-week-old wild-type or *Fmr1*-KO mice were received injections for 7 days before being sacrificed. Total proteins were extracted from the whole brain. (**A**–**D**) Representative Western blot images display the basal level of phosphorylated PKA (**A**) and GSK-3α/β (**C**) in female wild-type and *Fmr1*-KO mice treated only with saline (Wild-type: *n* = 4, *Fmr1*-KO: *n* = 4). Densitometric analyses of protein expression in wild-type animals were performed (**B**,**D**). The quantification of phosphorylated protein was normalized to total protein. (**E**–**H**) Representative Western blot images display the baseline level of phosphorylated PKA (**E**) and GSK-3α/β (**G**) in male wild-type and *Fmr1*-KO mice receiving saline only (Wild-type: *n* = 4, *Fmr1*-KO: *n* = 5). Densitometric analyses of protein expression in wild-type animals were performed (**F**,**H**). The quantification of phosphorylated protein was normalized to total protein. Data are presented as Mean ± SEM, ns—no statistical significance, (Student’s *t*-test, Shapiro-Wilk test was used to confirm the normality of the data.).

We compared the phosphorylation levels of PKA and GSK-3 between wild-type and *Fmr1*-KO animals. Female *Fmr1*-KO mice had significantly reduced phosphorylated PKA (Student’s *t*-test, *p* = 0.0003, *t* = 7.363, *df* = 6) (Figure 1A,B) and GSK-3, including both α (Student’s *t*-test, *p* = 0.0003, *t* = 7.363, *df* = 6) and β (Student’s *t*-test, *p* = 0.0004, *t* = 7.058, *df* = 6) subtypes (Figure 1C,D). Similarly, in male *Fmr1*-KO mice, phosphorylated PKA was significantly reduced (Student’s *t*-test, *p* = 0.0017, *t* = 7.058, *df* = 6) (Figure 1E,F). Although there was a trend towards reduced phosphorylated GSK-3α in male *Fmr1*-KO mice, this was not statistically significant (Student’s *t*-test, *p* = 0.19, *t* = 1.448, *df* = 7) (Figure 1G,H).

### 3.2. Sex Differences in Phosphorylated PKA and GSK-3β Are Lost in Fmr1-KO Mice

**Figure 2:**
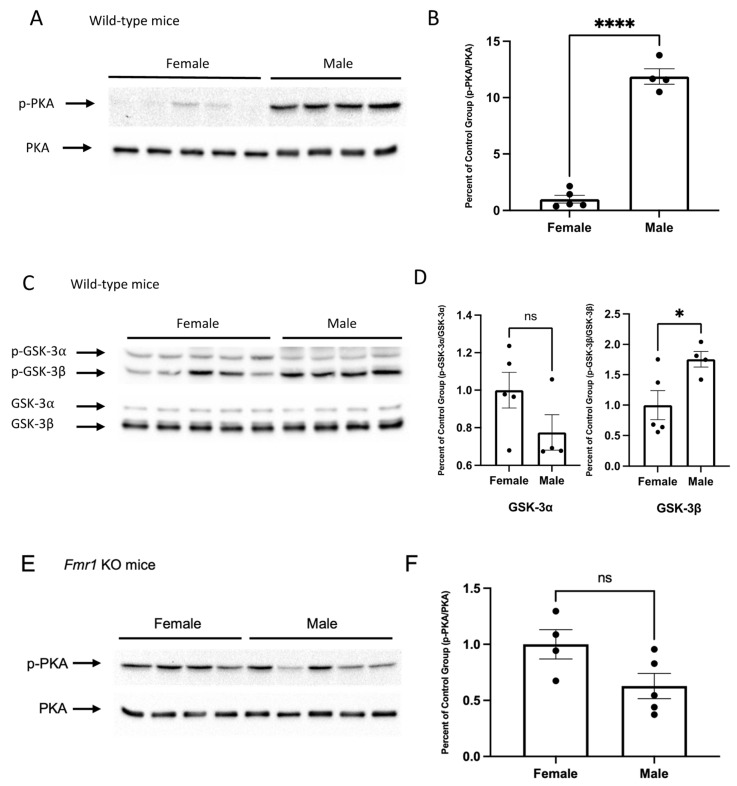

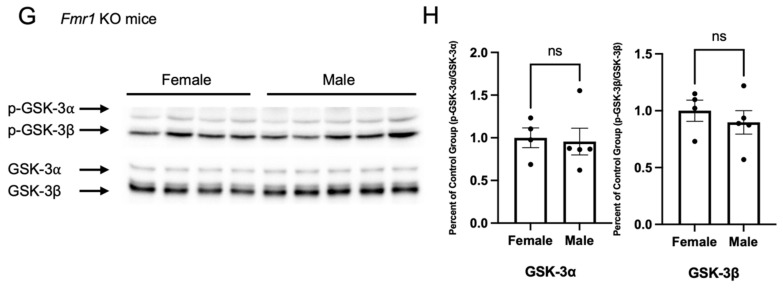
Sex differences in PKA and GSK-3 phosphorylation in wild-type and *Fmr1*-KO mice. Ten-week-old wild-type or *Fmr1*-KO mice received injections for 7 days before being sacrificed. Total proteins were extracted from whole brain. (**A**–**D**) Representative Western blot images display the basal level of phosphorylated PKA (**A**) and GSK-3α/β (**C**) in wild-type male and female mice treated only with saline (Female: n = 5, Male: n = 4). Densitometric analyses of protein expression in wild-type animals were performed (**B**,**D**). The quantification of phosphorylated protein was normalized to total protein. (**E**–**H**) Representative Western blot images display the baseline level of phosphorylated PKA (**E**) and GSK-3α/β (**G**) in *Fmr1*-KO male and female mice receiving only saline (Female: n = 5, Male: n = 4). Densitometric analyses of protein expression in wild-type animals were performed (**F**,**H**). The quantification of phosphorylated protein was normalized to total proteins. Data are presented as Mean ± SEM, ns - no statistical significance, * *p* < 0.05, **** *p* < 0.0001 (Student’s *t*-test, Shapiro-Wilk test was used to confirm the normality of the data.).

We quantified phosphorylated PKA (Thr-197) in wild-type mice under normal condition and found significantly higher levels of phosphorylated PKA in males compared to female mice (Student’s *t*-test, *p* < 0.0001, *t* = 15.23, *df* = 7) (Figure 2A,B). Similarly, the level of phosphorylated GSK-3β at Ser-9 was significantly higher in male wild-type mice compared to females (Student’s *t*-test, *p* = 0.0365, *t* = 2.579, *df* = 7). There were no significant sex differences in phosphorylated GSK-3α at Ser-21 in wild-type mice (Student’s *t*-test, *p* = 0.1416, *t* = 1.657, *df* = 7) (Figure 2C,D). In contrast, *Fmr1*-KO mice did not have significant sex differences in the level of p-PKA (Student’s *t*-test, *p* = 0.067, *t* = 2.166, *df* = 7) (Figure 2E,F), nor phosphorylated GSK-3β (Student’s *t*-test, *p* = 0.4966, *t* = 0.7171, *df* = 7) (Figure 2G,H).

### 3.3. L-Stepholidine Has Different Effects on Male and Female Wild-Type Mice

**Figure 3:**
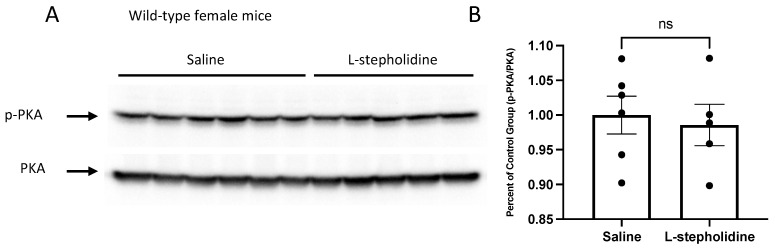

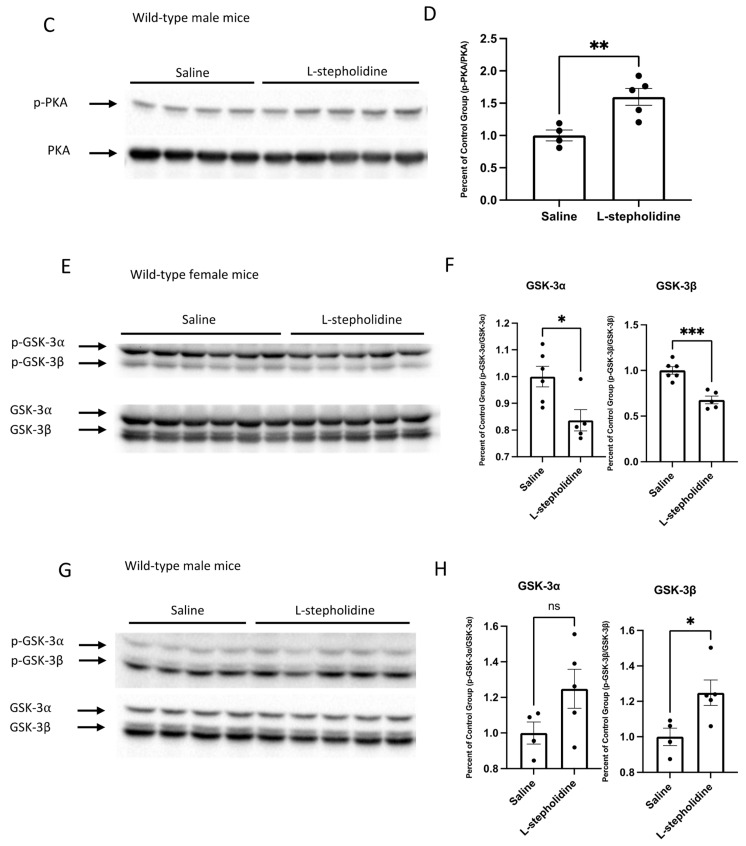
L-stepholidine had different effects on PKA and GSK-3 phosphorylation in male and female wild-type mice. Ten-week-old wild-type or *Fmr1*-KO mice received injections for 7 days before being sacrificed. Total proteins were extracted from whole brain. (**A**–**D**) Representative Western blot images show the level of phosphorylated PKA (**A**,**C**) in wild-type mice treated with saline or L-stepholidine (Female: Saline *n* = 6, L-stepholidine *n* = 5; Male: Saline *n* = 4, L-stepholidine *n* = 5). Densitometric analyses of protein expression of PKA (**B**,**D**) in wild-type mice were performed. The quantification of phosphorylated protein was normalized to total protein. (**E**–**H**) Representative Western blot images showing the level of phosphorylated GSK-3 (**E**,**G**) in wild-type mice treated with saline or L-stepholidine (Female: Saline *n* = 6, L-stepholidine *n* = 5; Male: Saline *n* = 4, L-stepholidine *n* = 5). Densitometric analyses of protein expression of GSK-3 (**F**,**H**) in wild-type mice were performed. The quantification of phosphorylated protein was normalized to total proteins. Data are presented as Mean ± SEM, ns—no statistical significance, * *p* < 0.05, ** *p* < 0.01, *** *p* < 0.001 (Student’s *t*-test, Shapiro-Wilk test was used to confirm the normality of the data.).

There was no change in phosphorylated PKA in female wild-type mice after daily injections of 10 mg/kg L-stepholidine for one week (Figure 3A,B). In contrast, male mice had a significant increase of phosphorylated PKA (Student’s *t*-test, *p* = 0.0086, *t* = 3.614, *df* = 7) (Figure 3C,D). L-stepholidine decreased phosphorylated GSK-3α and GSK-3β in female wild-type mice (GSK-3α: Student’s *t*-test, *p* = 0.0164, *t* = 2.942, *df* = 9; GSK-3β: Student’s *t*-test, *p* = 0.0004, *t* = 5.500, *df* = 9) (Figure 3E,F). Phosphorylated GSK-3β was significantly increased by L-stepholidine in male wild-type mice, and al-though phosphorylated GSK-3α was increased, this was not statistically significant (GSK-3α: Student’s *t*-test, *p* = 0.1099, *t* = 1.831, *df* = 7; GSK-3β: Student’s *t*-test, *p* = 0.0303, *t* = 2.708, *df* = 7) (Figure 3G,H).

### 3.4. L- Stepholidine Has Different Effects in Male and Female Fmr1-KO Mice

**Figure 4:**
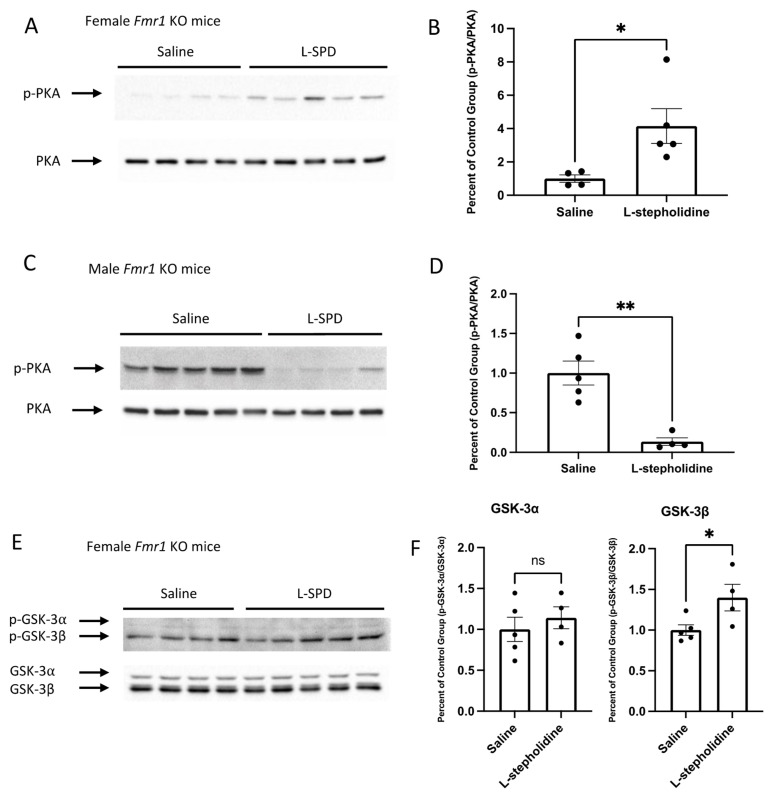

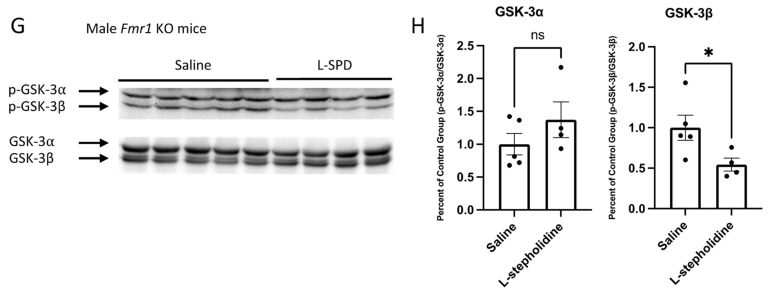
L-stepholidine had different effects on PKA and GSK-3 phosphorylation in male and female *Fmr1*-KO mice. Ten-week-old wild-type or *Fmr1*-KO mice received injections for 7 days before being sacrificed. Total proteins were extracted from whole brain. (**A**–**D**) Representative Western blot images display the level of phosphorylated PKA (**A**,**C**) in *Fmr1*-KO mice treated with either saline or L-stepholidine (Female: Saline *n* = 4, L-stepholidine *n* = 5; Male: Saline *n* = 5, L-stepholidine *n* = 4). Densitometric analyses of protein expression of PKA (**B**,**D**) in *Fmr1*-KO mice were performed. The quantification of phosphorylated protein was normalized to total protein. (**E**–**H**) Representative Western blot images display the level of phosphorylated GSK-3 (**E**,**G**) in *Fmr1*-KO mice treated with saline or L- L-stepholidine (Female: Saline *n* = 4, L-stepholidine *n* = 5; Male: Saline *n* = 5, L-stepholidine *n* = 4). Densitometric analyses of protein expression of GSK-3 (**F**,**H**) in *Fmr1*-KO mice were performed. The quantification of phosphorylated protein was normalized to total proteins. Data are presented as Mean ± SEM, ns - no statistical significance, * *p* < 0.05, ** *p* < 0.01 (Student’s *t*-test, Shapiro-Wilk test was used to confirm the normality of the data.).

L-stepholidine significantly increased PKA phosphorylation in female *Fmr1*-KO mice (Student’s *t*-test, *p* = 0.0336, *t* = 2.636, *df* = 7) (Figure 4A,B), while total PKA was unchanged. In contrast, phosphorylated PKA was significantly reduced by L-stepholidine in male *Fmr1*-KO mice (Student’s *t*-test, *p* = 0.0017, *t* = 4.908, *df* = 7) (Figure 4C,D). L-stepholidine increased GSK-3β phosphorylation (Student’s *t*-test, *p* = 0.0426, *t* = 2.474, *df* = 7) in female *Fmr1*-KO mice but had no significant effect on p-GSK-3α (Figure 4E,F). Male *Fmr1*-KO mice had reduced p-GSK-3β after receiving L-stepholidine, but there was no significant effect on p-GSK-3α (Student’s *t*-test, *p* = 0.0481, *t* = 2.391, *df* = 7) (Figure 4G,H).

## 4. Discussion

We sought to expand knowledge on dopamine signaling and function in FXS by examining sex differences in the phosphorylated PKA and GSK-3 response to L-stepholidine in wild-type and *Fmr1-*KO mice. We found sex differences in p-PKA and p-GSK-3β in wild type mice but not *Fmr1*-KO mice. L-stepholidine had opposite effects in wild-type mice compared to *Fmr1*-KO mice on these dopamine signaling components. L-stepholidine increased p-PKA and p-GSK-3β in male wild-type mice and decreased p-GSK-3 in female wild-type mice. In contrast, L-stepholidine increased p-PKA in female *Fmr1-*KO mice, but decreased p-PKA in male *Fmr1-*KO mice. This drug also increased p-GSK-3β in female *Fmr1-*KO mice while decreasing p-GSK-3β in male *Fmr1-*KO mice. Overall, we observed many opposing effects of the *Fmr1-*KO and stepholidine on intracellular dopamine signals in male and female animals.

Our results confirm and extend the literature on sex differences associated with FXS in humans and in *Fmr1*-KO mice. The complex pattern of changes in dopamine system-related phenotypes is consistent with the combined effects of a sex-linked mutation (*Fmr1* gene deletion) combined with more general sex differences in the dopamine system. For example, PET studies revealed greater D2-like receptor levels in the frontal cortex of women vs. men [36], with further sex differences in the regulation of dopamine release in the striatum [37]. The D1-D2 receptor complex is also observed at higher density in female vs. male rodents and non-human primates [38].

The sex differences we observed in *Fmr1*-KO mice are consistent with the many sex differences reported in human FXS patients. For example, adolescent male FXS patients have a greater cerebral volume than female patients [39], consistent with a larger volume of grey matter, cortical grey matter and the caudate nucleus in boys with FXS children [39]. Boys with FXS also tend to perseverate in speech more than girls [40]. Male FXS patients are more likely than females to have epilepsy [41]. Two antipsychotics, aripiprazole and Risperdal are also commonly used for ameliorating severe behavioural abnormalities patients such as irritability associated with aggression in male adolescents FXS [5,42,43,44]. These examples are not a comprehensive list, but serve to illustrate the variety of sex differences in brain structure and function caused by mutations in the *Fmr1* gene.

## 5. Conclusions

Overall, our results produced a complex picture of sex differences in dopamine signaling components and response to medications that are associated with *Fmr1* gene knock-out. While we did not investigate the mechanisms underlying these observations, they suggest that further research into these results could help to understand the clinical differences between male and female FXS patients. Our findings also suggest that there could be sex differences in responses to medication used in the management of patients with FXS, and this certainly warrants additional investigation [45]. Our experiments also reinforce that the *Fmr1* gene has a broad range of functions in the brain, and that disparate aspects of neurotransmitter signaling can be affected by the insufficiency of the FMR protein. These pleiotropic effects could be relevant to the wide range of clinical severity and manifestations of FXS, which complicates management of this complex disorder.

## Data Availability

Data reported from this paper are not posted in a publicly accessible database but are available from the authors.
